# Long-term avoidance memory formation is associated with a transient increase in mushroom body synaptic complexes in leaf-cutting ants

**DOI:** 10.3389/fnbeh.2015.00084

**Published:** 2015-04-08

**Authors:** Agustina Falibene, Flavio Roces, Wolfgang Rössler

**Affiliations:** Department of Behavioral Physiology and Sociobiology, Biozentrum, University of WürzburgWürzburg, Germany

**Keywords:** *Acromyrmex ambiguus*, leaf-cutting ants, avoidance learning, olfaction, synaptic plasticity, mushroom body, microglomeruli

## Abstract

Long-term behavioral changes related to learning and experience have been shown to be associated with structural remodeling in the brain. Leaf-cutting ants learn to avoid previously preferred plants after they have proved harmful for their symbiotic fungus, a process that involves long-term olfactory memory. We studied the dynamics of brain microarchitectural changes after long-term olfactory memory formation following avoidance learning in *Acromyrmex ambiguus*. After performing experiments to control for possible neuronal changes related to age and body size, we quantified synaptic complexes (microglomeruli, MG) in olfactory regions of the mushroom bodies (MBs) at different times after learning. Long-term avoidance memory formation was associated with a transient change in MG densities. Two days after learning, MG density was higher than before learning. At days 4 and 15 after learning—when ants still showed plant avoidance—MG densities had decreased to the initial state. The structural reorganization of MG triggered by long-term avoidance memory formation clearly differed from changes promoted by pure exposure to and collection of novel plants with distinct odors. Sensory exposure by the simultaneous collection of several, instead of one, non-harmful plant species resulted in a decrease in MG densities in the olfactory lip. We hypothesize that while sensory exposure leads to MG pruning in the MB olfactory lip, the formation of long-term avoidance memory involves an initial growth of new MG followed by subsequent pruning.

## Introduction

Social insects such as ants and honeybees are well known for their complex behavioral repertoires that include significant behavioral changes related to age, social environment and experience, together with impressive cognitive capabilities (Leadbeater and Chittka, 2007; Menzel et al., 2007; Giurfa, 2013). Ants are able to associate odors with positive rewards or negative outcomes, and they subsequently use this information during food collection (Roces, 1990, 1994; Dupuy et al., 2006; Provecho and Josens, 2009; Saverschek and Roces, 2011). Leaf-cutting ants, for example, learn to avoid certain food substrates (Knapp et al., 1990), particularly those that are harmful for their symbiotic fungus (Ridley et al., 1996; North et al., 1999; Herz et al., 2008; Saverschek et al., 2010; Saverschek and Roces, 2011).

Leaf-cutting ants harvest fragments from different plants and transport them to the nest to be used as substrate for rearing a symbiotic fungus, the sole food source of the developing brood (Quinlan and Cherrett, 1979; Bass and Cherrett, 1995; Bass, 1997). When ants incorporate a substrate into the fungus garden that contains compounds harmful to the fungus, forager ants subsequently stop harvesting from this substrate as a response to its negative effects on the fungus, a process called “delayed rejection” (Ridley et al., 1996; North et al., 1999; Herz et al., 2008; Saverschek et al., 2010; Thiele et al., 2014). Although ant workers may associate olfactory, gustatory and tactile stimuli from the incorporated substrate with the plant’s effects on the fungus, perception of the plant odor was shown to be sufficient for foraging workers to retrieve their memory about plant unsuitability for the fungus (Saverschek and Roces, 2011). Furthermore, this process involves long-term memory. Foragers recall avoidance memories when the plant species previously associated with the negative experience is presented after 9 weeks (in *Acromymrex lundi*) or even 16 weeks (in *Atta colombica*) after learning (Herz et al., 2008; Saverschek et al., 2010).

Long-term behavioral changes related to learning and experience have been shown to be associated with structural remodeling in the brain in both vertebrates and invertebrates (Kolb and Whishaw, 1998). This remodeling involves the rearrangement of synaptic contacts, including the formation of new synapses and the preservation, strengthening or elimination of other ones (Moser, 1999; Kantor and Kolodkin, 2003; Gogolla et al., 2007). In insects, learning and the formation of associative memories—like olfactory memories—have been shown to be associated with the mushroom bodies (MBs), higher order sensory integration and association centers in the insect brain (Erber et al., 1980; Menzel, 1990, 1999; Hammer and Menzel, 1995; Strausfeld et al., 1998; Heisenberg, 2003; Davis, 2005; Giurfa, 2007; Hourcade et al., 2010). In social Hymenoptera, the major MB sensory input regions (calyces) receive multimodal information segregated in different subregions: the lip receives input from olfactory projection neurons (PNs), whereas the collar is innervated by visual PNs (Gronenberg, 2001). In the MB calyx, PN presynaptic boutons contact Kenyon cell dendritic spines forming characteristic synaptic complexes called microglomeruli (MG; Ganeshina and Menzel, 2001; Gronenberg, 2001; Frambach et al., 2004; Groh et al., 2004, 2006; Seid and Wehner, 2008; Stieb et al., 2010, 2012).

The MBs were shown to exhibit significant changes associated with brood rearing conditions, behavioral transitions, age, and experience in ants (Gronenberg et al., 1996; Seid et al., 2005; Kühn-Bühlmann and Wehner, 2006; Seid and Wehner, 2009; Stieb et al., 2010, 2012), bees (Withers et al., 1993; Durst et al., 1994; Farris et al., 2001; Groh et al., 2004, 2012; Davis, 2005; Hourcade et al., 2010; Scholl et al., 2014), and wasps (O’Donnell et al., 2004). Each process appears to affect brain structure in different ways and these changes include variations in MB neuropil volumes and/or MG densities and numbers. In the context of associative learning, consolidation of a stable, transcription-dependent, olfactory long-term memory was shown to promote an increase in MG density in the lip region of the honeybee MB (Hourcade et al., 2010). This suggests that growth of new synaptic connections is involved in long-term storage of information, a mechanism that was also proposed in mammals (Moser, 1999; Lamprecht and LeDoux, 2004). However, the dynamics of these processes are not yet clear. For example, do increases in MG numbers associated with long-term memory formation remain stable over time? Do similar changes underlie the formation of positive and negative associations?

Considering the strength and enduring effect of plant avoidance learning in leaf-cutting ants, we studied the dynamics of changes in the neuronal microarchitecture of the main olfactory MB input regions after long-term memory formation following plant avoidance learning in *Acromyrmex ambiguus*. Recent studies on visual exposure in desert ants and honeybees showed that sensory exposure alone (exposure to light) leads to a reduction (pruning) in MG numbers in the visual (collar) region of the MB (Stieb et al., 2010; Scholl et al., 2014). To be able to distinguish between neuronal changes in the MB calyx triggered by learning vs. sensory exposure alone, we first investigated the effects following sensory exposure to novel plant odors in ants foraging on a large variety of plant species. As individual size and age are associated with structural changes in the MB in other social insects (Durst et al., 1994; Gronenberg et al., 1996; Groh et al., 2006, 2014; Kühn-Bühlmann and Wehner, 2006; Stieb et al., 2010), experiments to control for age- and size-related neuronal plasticity in* A. ambiguus* were also performed.

## Materials and Methods

### Animals

Experiments were conducted with subcolonies of *Acromyrmex ambiguus*. Compared with *Atta* leaf-cutting ants, this species has the advantage that relatively small subcolonies can rapidly be established and remain stable for several weeks. We used four different mother colonies, collected in Uruguay in 2005 (I) and 2008 (J, L, N), maintained in the laboratory under controlled conditions (25°C, 40–50% RH, 12:12 h light:dark cycle) as previously described (Saverschek and Roces, 2011). Approximately 500 cm^3^ of fungus garden (together with gardening workers) and 200 workers collected from the feeding chamber of one of the mother colonies were placed in an experimental nest consisting of three plastic boxes connected in a T-shaped fashion by plastic tubes (1 cm diameter, 8 cm length). Two large boxes (19 × 5 × 9 cm^3^) served as feeding and fungus chambers, while a smaller one (9 × 9 × 5 cm^3^) served as refuse chamber (Herz et al., 2008; Saverschek et al., 2010; Saverschek and Roces, 2011). Subcolonies were established at least 4 days prior to experiments and were provided daily with water, honey water and fresh blackberry (*Rubus fructicosus*) leaves, which were also used (together with rose leaves, *Rosa canina*) throughout the year for the maintenance of the laboratory colonies.

### Control Experiment One: Age-Related Neuronal Plasticity

Considering that changes in neuronal structures and MG numbers in the MB calyces were shown to be age related in several species of ants and in honeybees (Durst et al., 1994; Gronenberg et al., 1996; Groh et al., 2006; Kühn-Bühlmann and Wehner, 2006; Stieb et al., 2010), we first quantified age related changes in the microarchitectural organization of the MB calyx in *A. ambiguus* leaf-cutting ants. In order to control the age of individual ants, we used the method described by Camargo et al. (2007). Around 300 pupae of different sizes were collected from a single colony (colony I) and placed in a container box (19 × 9 × 5 cm^3^) with the base covered by a 1 cm layer of plaster to maintain humidity. About 100 adult workers from the same colony were isolated, paint-labeled (Edding 751 paint marker, Edding International GmbH, Ahrensburg, Germany) on the gaster, and placed together with the pupae to provide them care during growth and assistance during eclosion. Approximately 100 cm^3^ of fungus garden—free of workers—was added to the container to provide food for the workers. Every day at the same time, newly emerged ants were removed. One group was taken for dissection immediately after eclosion (<24 h), while the remaining ants were marked after eclosion using different colors every 2 days. Once marked, individuals were moved to a different subcolony (from the same mother colony) with unmarked workers. Newly emerged marked ants were kept there until they reached different ages. Brain dissection and presynaptic bouton staining (see below) were then performed for the following groups: *1-day old workers*: ants taken immediately after eclosion (<24 h), *1-week old workers*: ants taken 7–8 days after eclosion, 1-month-old *workers*: ants taken 28–29 days after eclosion, and *outside workers*: individuals of unknown age taken from the feeding chamber of the experimental subcolony. All ants dissected were of similar size: medium sized workers with a head width (HW) within a range of 1.2–1.5 mm.

### Control Experiment Two: Body-Size Related Neuronal Organization

*A. ambiguus* is a highly polymorphic species. Given that no information on worker size distribution for this species was available, we first addressed this issue. Individuals were collected from both the fungus and the feeding chamber (colony N), weighed (body mass, M, mg), and then anesthetized on ice and decapitated in order to measure the HW (mm). The relationship between M and HW was calculated and used to compute each individual’s mass as a non-invasive and simple body size measurement that allow us to select individuals of different size ranges.

We then investigated the relationship between worker size and MG densities and numbers in the MB calyces. Ants were taken from the feeding chamber of the nest to ensure that all were outside workers. We compared ants from two different size classes that correspond to the extremes of the foragers’ size range. It extended from 1.0 to 2.0 mm (HW, mean value: 1.50 ± 0.03 mm, *N* = 43), as determined in independent measurements. Ants were arbitrarily classified as medium (HW = 1.0–1.3 mm) and large (HW = 1.7–2.0 mm) workers. Individuals from both groups were then anesthetized on ice, decapitated, and their brains dissected for synaptic immunostaining (see below).

### Effects of Sensory Exposure

Experiments were performed using well-established, long-lasting laboratory colonies fed year-round with only one or two different plant species. In comparison with ants living in natural environments with high variability of plant species, workers from the laboratory colonies were therefore maintained under relatively deprived sensory conditions. During the learning experiments, ants became exposed to novel plant species. We therefore investigated whether sensory exposure to novel odors of leaves from multiple plant species alone may lead to MG reorganization in the olfactory input region of the MB calyces. To address this question, two subcolonies from the same mother colony (J) were established at the same time and offered leaves of a different number of plant species in the experimental arena during three consecutive days (days 0, 1, 2). Leaves were offered in the feeding chamber as discs of 7 mm in diameter cut out of fresh leaves. While one subcolony received leaves of only one plant species (blackberry, 100 discs each day), the other subcolony was offered leaves from 10 different plant species: blackberry, scarlet firethorn * (Pyracanta coccinea)*, privet * (Ligustrum sp.)*, broccoli* (Brassica oleracea*), coleus* (Solenostemon sp*.), cashew* (Anacardium occidentale*), cotoneaster* (Cotoneaster sp*.), mediterranean buckthorn* (Rhamus alaternus*), mango* (Mangifera indica*) and citrus (*Citrus sp*.); 10 discs of each species each day. On day 3, workers collecting discs from the experimental arena were captured on the bridge when returning to the nest, and their brains dissected for immunostaining (see below). In this subcolony, the size of the foragers was slightly larger than in previous experiments, but still within the range previously observed for foragers in another colony (i.e., HW: 1.0–2.0 mm). In order to get a higher number of individuals, foragers with HW between 1.7 and 1.8 mm were selected from both subcolonies and compared between treatments.

### Effects of Long-Term Avoidance Memory Formation

Learning was established by offering ants plant leaves that are initially accepted by them but then turn out to be unsuitable for the symbiotic fungus, as previously reported (Herz et al., 2008; Saverschek et al., 2010; Saverschek and Roces, 2011). In order to change the suitability of the substrate, the internal air space of discs cut out of fresh leaves (discs of 7 mm in diameter) was infiltrated with a 0.03% w/w aqueous solution of cycloheximide (CHX; Sigma-Aldrich, Deisenhofen, Germany) by a pressure-vacuum infiltration method (Beyschlag and Pfanz, 1990; Herz et al., 2008). Then, infiltrated discs were briefly rinsed with water and blotted dry with paper tissue. CHX acts as a fungicide and has previously been proven to be undetectable by—and not harmful to—the ants, but harmful to the attine symbiotic fungus (Ridley et al., 1996; North et al., 1999; Herz et al., 2008).

We performed two different learning experiments as described below. The first one explored neuronal remodeling over 4 days after the colonies were offered treated leaves twice. In the second learning experiment, treated leaves were offered only once to more precisely time the experiment onset, and responses were followed over a period of 15 days. Experiments were done at different times and by using subcolonies from different mother colonies. Two subcolonies were used for each experiment: one of them was offered leave discs infiltrated with CHX (treatment) while the other one was offered leave discs without any treatment (control). As behavioral responses and brain synaptic organization may depend on multiple factors and potentially vary between colonies, comparisons were performed among individuals from the same subcolony (each ant was considered as an experimental unit) before and at different times after the incorporation of the leaves into the fungus garden.

#### Learning Experiment 1

Two different subcolonies were set from colonies L and N and treated independently. On the initial day of the experiment (day 0), the nest feeding chamber was connected to the experimental feeding arena (19 × 9 × 5 cm^3^) by a wooden bridge (120 cm). In order to guarantee a well-established foraging column, ants were allowed to forage for about 30 min to collect ~20 discs of fresh blackberry leaves. Afterwards, a choice experiment (preference test) to test for any initial plant preferences started. The ants had to choose between two different plant species: privet and scarlet firethorn. Ten fresh discs of each species (without any treatment) were offered in the experimental arena. During the next 100–120 min, each ant carrying a disc was captured on the bridge when returning to the nest and kept separately from the subcolony. The forager’s decision was recorded, and the collected disc replaced by a fresh one. Leaves were not incorporated into the fungus garden during the test. All plant species used were collected every day at the university campus. These plants were previously known to be accepted and incorporated into the fungus garden by *A. ambiguus* laboratory colonies. In the initial preference tests, all foragers were captured irrespective of their choice. Those having HWs between 1.2 and 1.5 mm were selected, and their brains dissected for synaptic labeling (see below). Subsequently, subcolony assigned to the treated group (from colony N) received approximately 100 CHX-infiltrated discs of firethorn leaves on two consecutive days (day 0 and 1), each day. Control subcolony (from colony L) received 100 untreated discs of the same plant each day. To assess long-term memory, the avoidance of the unsuitable plant was tested. For that, a preference test was repeated 1, 2 and 4 days after the incorporation of the leaves into the fungus garden following the same procedure described before. As previously done on day 0, medium-sized foragers (HW: 1.2–1.5 mm) were selected after the preference tests on days 2 and 4, irrespective of their choice, and their brains dissected for synaptic labeling (see below). Since the treated leaves were offered to the whole subcolony, and consistent colony-wide responses are known to occur (Saverschek and Roces, 2011), we assumed that all workers were able to associate the contingency, even those that accepted the plant that should be avoided in a single preference test.

#### Learning Experiment 2

Two new subcolonies were set from colonies I and L. After the initial preference test, one of them (treated, from colony L) was offered 100 CHX-infiltrated discs of firethorn leaves on day 0, while the other one (control, from colony I) was offered 100 untreated discs of the same plant. Long-term memory was assessed over a longer period of time, as compared to the learning experiment 1, by performing further preference tests 1, 2, 4 and 15 days after the incorporation of the leaves into the fungus garden. After the preference tests on days 0, 2, 4 and 15, medium-sized foragers (HW: 1.2–1.5 mm) were selected, and their brains were dissected for synaptic labeling (see below).

### Immunostaining of Microglomeruli, Laser-Scanning Confocal Microscopy, Image Processing, and Data Acquisition

To analyze structural organization in the MB calyces, we used a well-established protocol for immunolabeling of presynaptic terminals of whole mount preparations (Groh et al., 2012, 2014). By immunolabeling synapsin –a protein associated with synaptic vesicles– in whole-mounts we were able to measure the volume of the MB calyces and, in the same preparation, quantify individual MGs by visualizing the synapsin-immunoreactive (IR) large presynaptic boutons of PNs. Ants were anesthetized on ice and decapitated. Heads were fixed in dental-wax coated dishes. Brains were dissected in cold Ringer solution and immediately transferred to a cold fixative solution (4% Formaldehyde in phosphate-buffered saline, PBS, pH 7.2) to be fixed overnight at 4°C. Brains were then washed in 0.1M PBS (3 × 10 min), then in PBS containing 2% Triton-X 100 (1 × 10 min) and finally in PBS with 0.2% Triton-X 100 (PBS-0.2%T, 2 × 10 min). Afterwards, they were pre-incubated in PBS-0.2%T with 2% normal goat serum (NGS; ICN Biomedicals, No. 191356, Orsay, France) for 1 h at room temperature. Preparations were then incubated in a mouse monoclonal antibody to the *Drosophila* synaptic-vesicle-associated protein synapsin I (1:10; SYNORF1, kindly provided by Dr. E. Buchner, University of Würzburg, Germany) in PBS-0.2%T with 2% NGS for 4 days at 4°C. Afterwards, brains were washed in PBS (5 × 10 min) and incubated in CF488A goat anti-mouse secondary antibody (1:250, Biotium) in PBS with 1% NGS for 2 days at 4°C. After washing in PBS (3 × 10 min), post-staining fixation was performed overnight at 4°C. Brains were then washed in PBS (5 × 10 min) and dehydrated through an ascending ethanol series (30%, 50%, 70%, 90%, 95%, 3 × 100%, 10 min per step). Finally, preparations were cleared and mounted in methyl salicylate (M-2047, Sigma Aldrich, Steinheim, Germany) using aluminum slides with a central hole covered from both sides by thin coverslips (Nr. 00: 55–80 μm, Menzel-Gläser, Germany).

Whole-mount preparations were visualized by using a laser-scanning confocal microscope (Leica TCS SP2, Leica Microsystems, Wetzlar, Germany) equipped with an argon/krypton laser. Optical sections were taken at a resolution of 1024 × 1024 pixels. For volume measurements of MB calyx subregions, optical sections were taken at 3 μm intervals through the entire left medial calyx using a 20 × 0.7 NA imm objective and a 3× digital zoom. For quantification of synapsin-IR boutons, optical sections of the innermost part of the left medial calyx were taken at 0.5 μm intervals through a depth of 10 μm using a 63 × 1.4 NA imm objective and a 2× digital zoom. Previous work revealed no differences in MG densities between the medial and lateral calyces, and within different areas of the lip subregions (Groh et al., 2004, 2014; Stieb et al., 2010).

Images were processed with 3D software (AMIRA v. 5.3; FEI Visualization Sciences Group, Düsseldorf, Germany) following well established procedures (Groh et al., 2004, 2006, 2012, 2014; Stieb et al., 2010; Groh and Rössler, 2011). For volume measurements of calyx subregions, the outer borders of the lip and collar were traced on each section, 3D reconstructed, and the volumes were calculated. In *Atta* leaf-cutting ants, the lip was shown to be subdivided into two different subregions: non-dense (ND, inner region) and dense (D, outer region) lip (Groh et al., 2014). To estimate the density of MG, we counted the synapsin-IR boutons within two different 1000 μm^3^ volumes (86 × 86 voxel) in the ND lip. In the age-related synaptic plasticity experiments, we additionally analyzed the bouton density in the D lip. In the learning experiments, to control for possible changes in MG densities which are not related with olfactory memory formation, we also analyzed synapsin-IR bouton densities in the visual collar of ants from the treated group (*learning experiment 2*). In *A. ambiguus*, the collar and the D lip subregion are smaller than the ND lip. In these regions, synapsin-IR boutons were then counted within two different 486.74 μm^3^ volumes (60 × 60 voxel) selected in each region of interest. For each individual, bouton numbers were averaged separately for each region and bouton density calculated as number of boutons per μm^3^.

### Statistical Analyses

Volumetric variables and bouton densities were compared among groups by using one-way ANOVA analyses followed by *post-hoc* Tukey comparisons when necessary. Normality and homogeneity of variance assumptions were tested in all cases. When these assumptions were not met, a Kruskal–Wallis ANOVA test was used. In the learning experiments, statistically significant ANOVA tests were followed by *post-hoc* Dunnett’s tests (comparison of day 0—initial state—to each other group). In control experiment one, where bouton densities were compared for the different subregions of the lip and across ages, a two-way ANOVA for repeated measures (RM-ANOVA) was used (age factor: 4 levels; lip subregion factor—repeated measure: 2 levels). Ants’ preferences in the learning experiments were analyzed by using *G*-tests. The significance level used was 5% in all cases.

## Results

### Control Experiments for Age and Body Size

#### Age-Related Differences in MB Calyx Synaptic Organization

**Figure 1:**
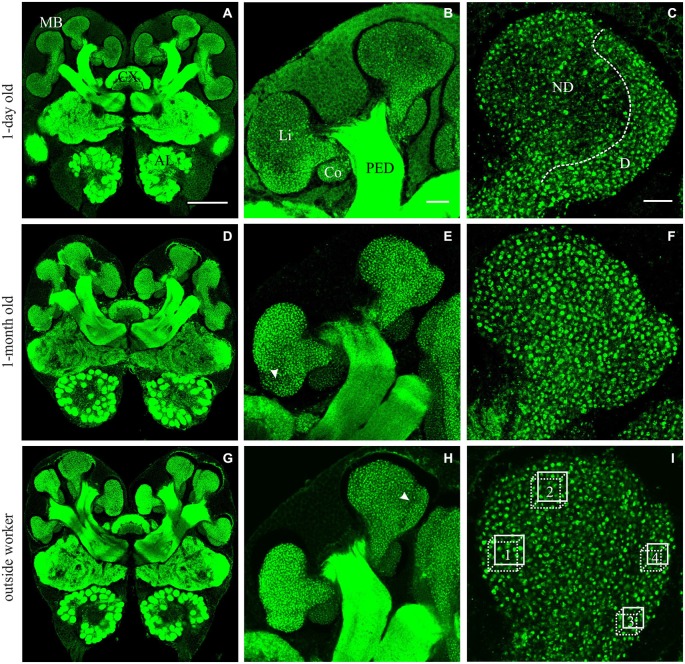
**Immunofluorescence staining with anti-synapsin in brains from *A. ambiguus* workers of different ages: examples of 1-day old (A–C), 1-month old (D–F), and from outside-workers of unknown age (G–I). (A,D,G)** Frontal overview of brains in a central plane. mushroom bodies (MBs), central complex (CX) and antennal lobes (AL) are indicated. **(B,E,H)** Comparison of the staining of MB calyces at different ages. Overview of the left medial calyx: lip (Li), collar (Co) and peduncle (PED). **(C,F,I)** Higher magnification of the lip calycal region. In 1-day old ants, the lip appeared differentially stained. The non-dense (ND) lip presented lower number of synapsin-immunoreactive (IR) boutons compared with the dense (D) lip. In older ants, in which the boutons clearly appeared as well defined structures, this differentiation was not obvious and two subregions could be defined by an unstaining band between them—as was previously reported for other ant species (Gronenberg, 2001)–, indicated by the arrow in **(E,H)**. In **(I)**, boxes 1–2 and 3–4 indicate the volumes used for quantification of MGs in the ND and D subregions of the MB calyces, respectively. Scale bars: in **(A)** (valid for **A,D,G**): 100 μm; in **(B)** (valid for **B,E,H**): 20 μm; in **(C)** (valid for **C,F,I**): 10 μm.

**Figure 2:**
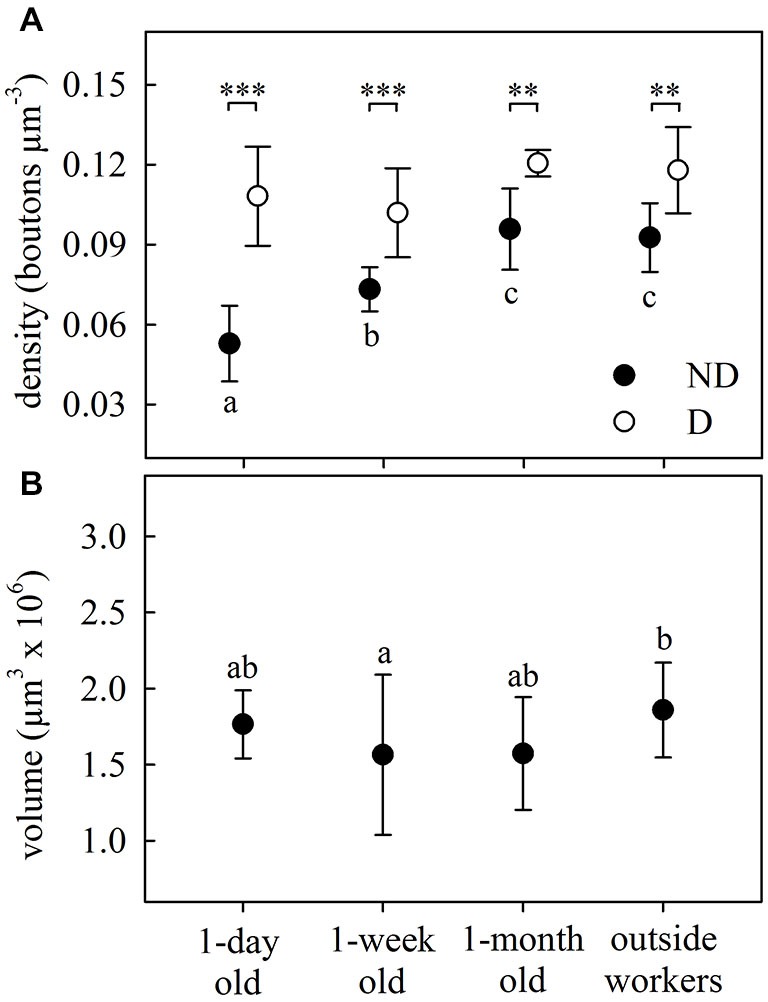
**Age-dependent structural plasticity in the MB calyces. (A)** Bouton density in both the ND and D lip subregions of the MB calyces. Number of boutons per area increased with age in the ND lip and remained constant in the D lip, where the overall density was higher. **(B)** Total lip volume varied with ant age. Dots represent the mean value of each group and lines the S.D. Asterisks indicate significant differences between lip subregions (ND vs. D lip): ** *p* < 0.01; *** *p* < 0.001. Different letters indicate significant differences in ND lip or lip volume among ages. One-day old, *N* = 9; 1-week old, *N* = 8; 1-month old, *N* = 5; outside-workers, *N* = 6.

Age-related differences were found in the olfactory lip region of the MB in *A. ambiguus* (Figure 1). In 1-day old workers, the inner subregion of the lip (ND lip) showed an overall weaker staining compared to the outer subregion (D lip) (Figures 1A–C). These differences were less obvious in older individuals (Figures 1D–I). The density of synapsin-IR boutons in the ND lip was lower than in the D lip for all age groups (age *x* lip-subregion: *F*_3,23_ = 5.15, *p* = 0.007. Lip-subregion simple effect: *p* ≤ 0.005, for all ages; RM-ANOVA; Figure 2A). This is in accordance with the results found in *A. vollenweideri* leaf-cutting ants of unknown ages (Groh et al., 2014). Boutons continuously increased in density with age from 1-day to 1-month old workers specifically in the ND lip (age in ND lip simple effect: *F*_3,46_ = 13.44, *p* < 0.0001; RM-ANOVA). The ND lip in one-day old ants, on average, contained only 57% of the boutons found in 1-month old ants, and 1-week after eclosion this number rose to 86%. The differences between 1-month old ants and outside workers, however, were no longer significant. Here, no significant age-related difference was found for the D lip density (age in D lip simple effect: *F*_3,46_ = 2.20, *p* = 0.10; RM-ANOVA).

We then analyzed how variations in bouton densities were associated with volumetric changes of the MB calyces. The total lip volume showed significant variations (*F*_3,23_ = 3.48, *p* = 0.032; ANOVA; Figure 2B), but not clear-cut relationship with age. There were no differences from 1-day to 1-month old workers, and only the lip volume of outside workers was significantly higher than that of 1-week old workers. This suggests that the overall increase in bouton density in the lip was due to an increase of the total number of MG rather than a lip volume reduction. The observed volumetric variation was modality-specific as no significant differences were found in the collar (*F*_3,24_ = 1.66, *p* = 0.20; ANOVA). However, as we did not analyze MG densities in the collar of age-controlled ants, we cannot rule out an age-related volume independent reorganization of MG in the visual collar of this ant species.

Considering that leaf-cutting ants start to participate in activities outside the nest by 3 or 4 weeks of age (Julian and Fewell, 2004; Camargo et al., 2007), we assume that the group defined as “outside workers” is composed of ants of different ages, but all of them should be older than 1 month. As no significant differences were found in the MB lip region between 1-month old ants and outside workers of unknown age (Figures 2A,B), we conclude that age-related variations did not occur under our laboratory conditions beyond 1-month post-emergence. Based on this result, and for reasons of experimental time, we decided not to control for age of foraging ants in the following experiments. In addition, bouton density quantification suggests that changes in the microstructure of the MB calyces are more likely to occur in the ND lip, while D lip appeared to show less plasticity. Consequently, we focused our analyses in the following experiments on the ND lip.

#### Body-Size-Related Differences in MB Calyx Synaptic Organization

**Figure 3:**
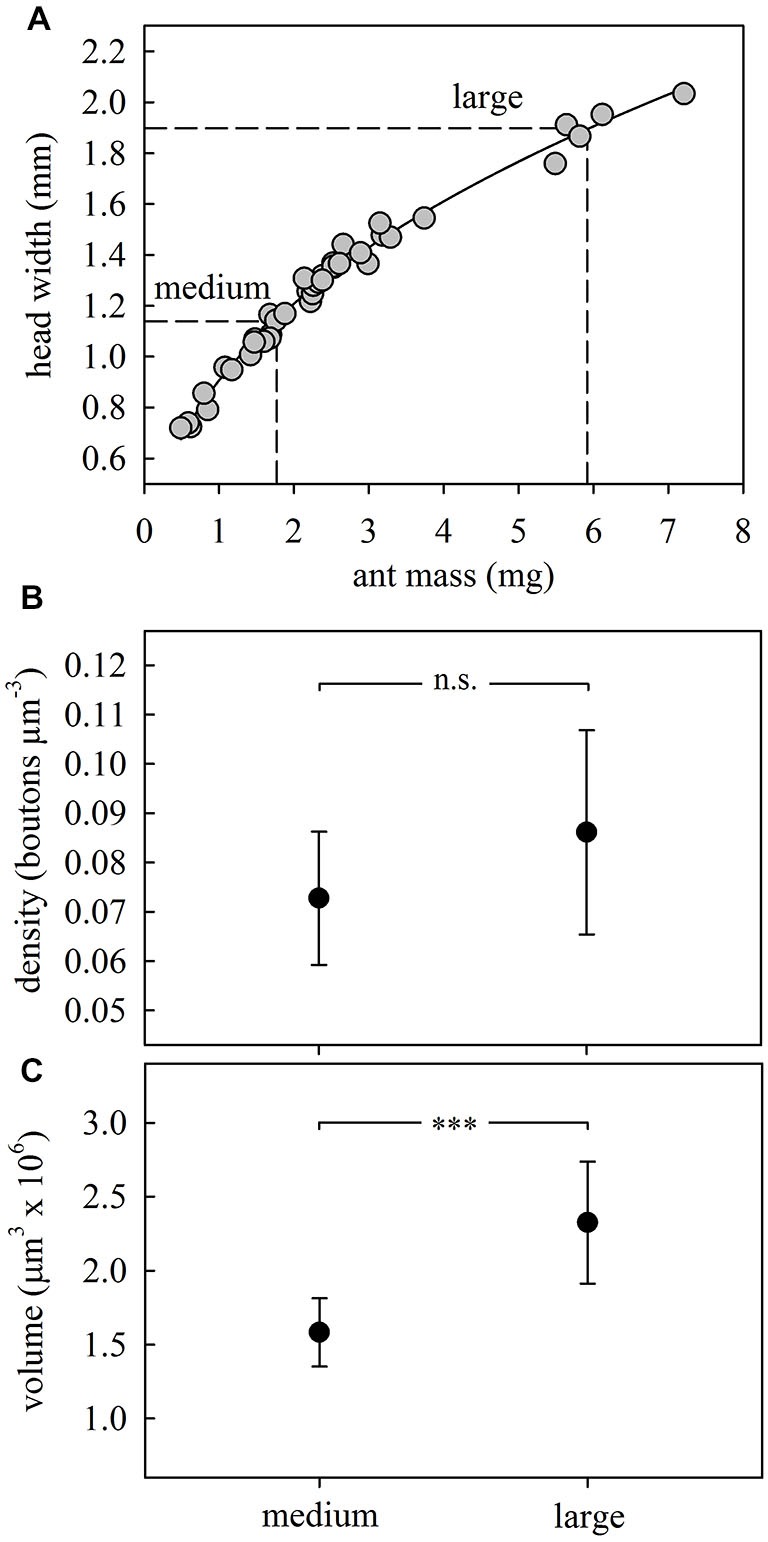
**Size-dependent synaptic organization in the MB calyces. (A)** Body morphometry for *A. ambiguus* workers. The following relationship was found between head width (HW) and body mass (M): HW = 0.9066*M^0.4139^; *R*^2^ = 0.99. To compare the MB structure between ants of different sizes we selected outside workers with sizes corresponding to both extremes of the foragers’ size range (HW: 1.0–2.0 mm), defining them as medium and large. Every dot represents an ant (*N* = 40). **(B)** Bouton densities remained constant between ants of different sizes. **(C)** Lip volume increased with ant size. In **(B,C)** dots represent the mean value and lines the S.D. ***p < 0.001. Medium, *N* = 8; large, *N* = 10.

Comparison of medium (HW = 1.14 ± 0.02 mm) and large (HW = 1.89 ± 0.02 mm) outside workers (Figure 3A) did not reveal significant differences in ND lip bouton densities (*F*_1,16_ = 2.46, *p* = 0.14; ANOVA; Figure 3B). However, both lip (*F*_1,16_ = 20.57, *p* = 0.0003; ANOVA; Figure 3C) and collar volumes (*F*_1,16_ = 19.91, *p* = 0.0004; ANOVA) increased with ant body size. Both results are in accordance with results reported for the highly polymorphic leaf-cutting ant *A. vollenweideri* (Groh et al., 2014). In the following experiments, we therefore analyzed brains of ant foragers of a similar range in size to exclude variability within and between groups.

### Effects of Sensory Exposure

**Figure 4:**
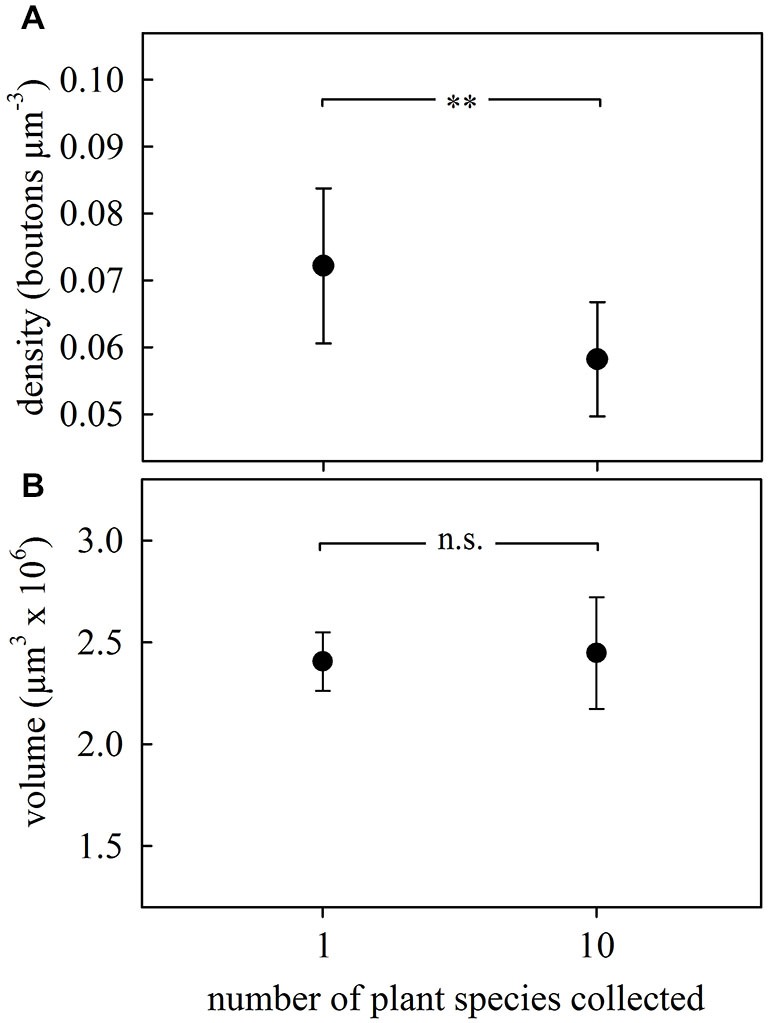
**Changes in the MB calyx synaptic organization promoted by odor sensory exposure by experiencing different kinds of leaves. (A)** Density of synapsin-IR boutons in the ND lip region decreased in the brains of ants from the subcolony exposed to a higher variety of plant species. **(B)** Lip volume did not change with the experience. Dots represent the mean value and lines the S.D. ** *p* < 0.01. 1 plant species, *N* = 9; 10 plant species, *N* = 10.

We investigated whether simultaneous collection and incorporation of multiple plant species with distinct odors (as it often occurs under natural situations) leads to changes in MG organization in the olfactory MB calyces. We compared ants from subcolonies that had foraged on a single plant species with those that had experienced 10 different kinds of leaves over three consecutive days. Although ants showed preferences for some of the species, none of them was rejected during the 3-day experiment (data not shown). The simultaneous exposure to and collection of several plant species promoted after 3 days a significant reduction (~20%) of synapsin-IR bouton densities in the ND lip of the MB calyces compared to ants exposed to only one plant species (*F*_1,17_ = 9.08, *p* = 0.008; ANOVA; Figure 4A). As there were no changes in lip volume between groups (*F*_1,17_ = 2.82, *p* = 0.11; ANOVA; Figure 4B), this suggests that the decrease in bouton densities was due to a net reduction in MG numbers. No significant differences were found in collar volumes (*F*_1,17_ = 3.21, *p* = 0.09; ANOVA).

### Changes in MG Related to Long-Term Memory Formation

#### Learning Experiment 1

**Figure 5:**
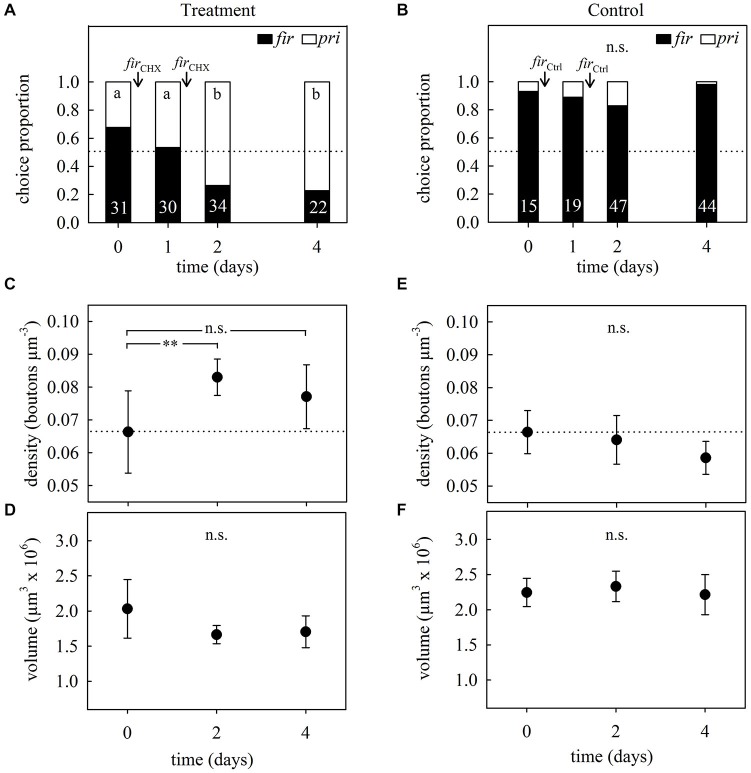
**Long-term memory assessment and associated changes in MB synaptic organization at different times after avoidance learning in 2-day treatment experiments. (A)** Foragers’ plant preferences for treated and **(B)** control subcolonies. Workers had to choose between firethorn (*fir*) and privet (*pri*) untreated leaf discs. During two consecutive days after the preference test they were offered treated (*fir*_CHX_) or untreated (*fir*_Ctrl_) firethorn leaves. Bars show the proportion of taken plant discs. Different letters indicate significant differences among days (G-Test) and numbers at the bottom of each bar indicate the total number of ants that collected a disc during testing. **(C)** Density of synapsin-IR boutons in the ND lip and **(D)** total lip volume at different times after the incorporation of CHX-infiltrated leaves (*fir*_CHX,_ treatment). ND lip density increased without volumetric changes of the lip after plant avoidance learning. **(E)** ND lip synapsin-IR boutons density and **(F)** lip volume at different times after incorporation of the untreated plant (*fir*_Ctrl,_ control). Incorporation of control leaves did not promote significant changes in the lip. Dots represent the mean value and solid lines the S.D. Horizontal dotted lines indicate mean bouton density quantified before treatment (day 0). Asterisks indicate significant differences between day 0 and different times after leaves incorporation into the fungus garden; ** *p* < 0.01. Treatment: day 0, density: *N* = 6, volume: *N* = 7; day 2, *N* = 6; day 4, density: *N* = 7, volume: *N* = 8. Control: day 0, *N* = 7; day 2, *N* = 7; day 4, *N* = 7.

Preference tests showed that foragers from both treated and control subcolonies had an initial preference for firethorn leaves (Figures 5A,B, day 0). Foragers from the treated subcolony—in which CHX-infiltrated firethorn leaves were incorporated—showed a clear rejection behavior from the second day after incorporation of the treated leaves into the fungus garden (hereafter referred to as “incorporation”) (GH = 16.95, *p* = 0.0007, *N* = 117, *df* = 3; G-test; Figure 5A). On the other hand, workers from the control subcolony did not change their preference after incorporation of the untreated leaves (GH = 6.48, *p* = 0.09, *N* = 125, *df* = 3; G-Test; Figure 5B). This means that the plant itself was not harmful for the fungus and was still preferred by the ants 4 days after its incorporation. We conclude that ants from the treated subcolony showed long-term avoidance memory evidenced by the rejection of the plant species previously associated with the CHX effects on the fungus.

**Figure 6:**
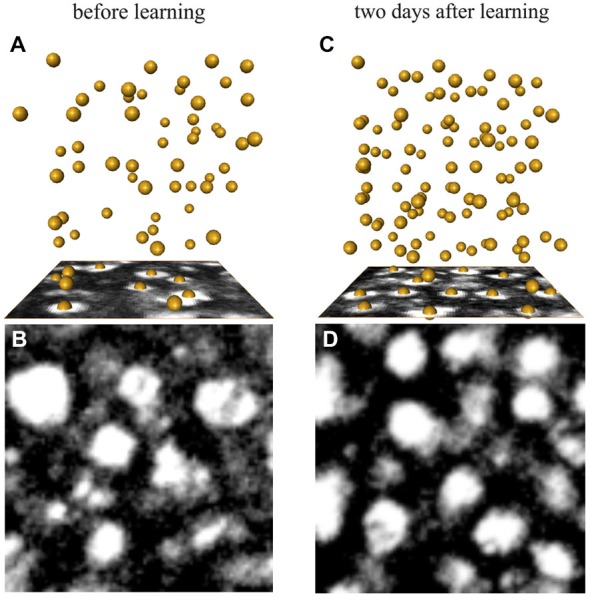
**Examples of synapsin-IR bouton quantification in the ND lip (A,B) before and (C,D) 2 days after learning. (A,C)** Three-dimensional reconstruction of the position of the boutons visualized by AMIRA in a 1000 μm^3^-volume (86 × 86 voxel). Each yellow sphere marks the center of a bouton. **(B,D)** Single confocal image of a 10 × 10 μm^2^ (86 × 86 voxel) synapsin-stained area in the ND lip.

Figure 5 further shows the analysis of the MG organization in the MB calyx lip before and at different times after long-term avoidance memory formation. Examples of synapsin-IR bouton quantifications before and 2 days after learning are shown in Figure 6. Two days after learning, the number of synapsin-IR boutons per volume in the ND lip was significantly higher compared to the initial number (25% increase compared to day 0; *F*_2,16_ = 4.44, *p* = 0.029; ANOVA; Figure 5C). Interestingly, synapsin-IR bouton densities in the lip region decreased on day 4, averaging intermediate values between days 0 and 2, yet not different from those of day 0. We then analyzed whether changes in synapsin-IR bouton densities were caused by a reduction in lip volume and found no significant volumetric changes in the lip (*F*_2,18_ = 3.41, *p* = 0.06; ANOVA; Figure 5D), nor in the collar (*H*_2, *N* = 21_ = 3.73, *p* = 0.15; Kruskal–Wallis). This suggests that the changes in synapsin-IR bouton densities were due to an increase in the number of synapsin-IR boutons in the ND lip.

As the sensory exposure to several novel plant species simultaneously resulted in an MG density decrease, as indicated above, we further tested whether sensory exposure to only one novel suitable plant species alone may promote changes in MG organization. Brains of foragers from the control subcolony that had collected firethorn leaves without any treatment during two consecutive days showed a slight but no significant decrease in synapsin-IR bouton densities after incorporation (*F*_2,18_ = 2.73, *p* = 0.10; ANOVA; Figure 5E). Neither the lip (*F*_2,18_ = 0.47, *p* = 0.63; ANOVA; Figure 5F) nor the collar (*F*_2,18_ = 1.23, *p* = 0.32; ANOVA) volume changed significantly. This indicates that the experience of incorporating one novel and suitable plant species to the fungus garden alone did not promote quantifiable changes in MB neuronal organization.

#### Learning Experiment 2

**Figure 7:**
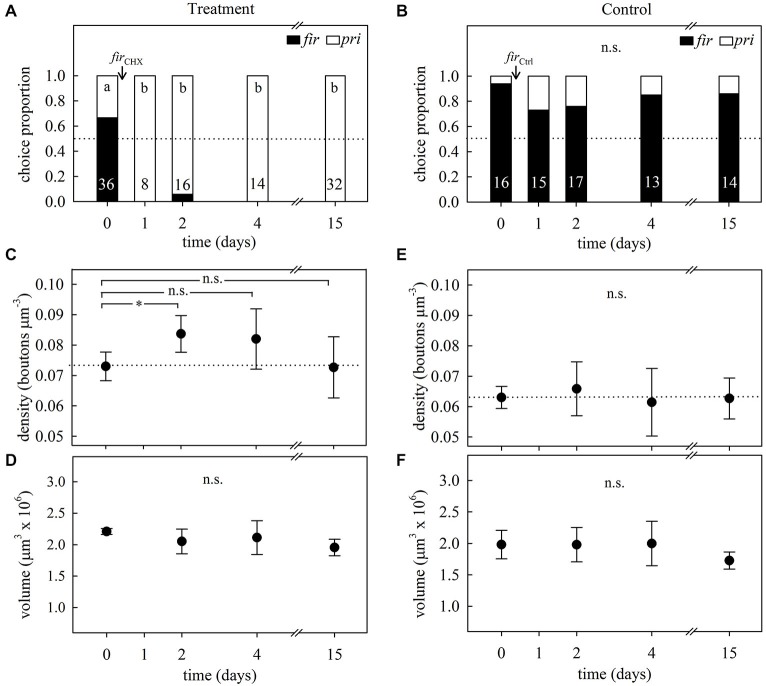
**Long-term memory assessment and its associated changes in MB synaptic organization at different times after avoidance learning in 1-day treatment experiments. (A)** Foragers’ plant preferences for treated and **(B)** control subcolonies in a choice experiment between firethorn (*fir*) and privet (*pri*) leaves. On day 0, ants were offered treated (*fir*_CHX_) or untreated (*fir*_Ctrl_) firethorn leaves. Bars show the proportion of taken plant discs at different times after treatment. Different letters indicate significant differences among days (G-Test) and numbers at the bottom of each bar indicate the total number of ants that collected a disc during testing. **(C)** Density of synapsin-IR boutons in the ND lip and **(D)** total lip volume at different times after the incorporation of CHX-infiltrated leaves (*fir*_CHX,_ treatment). Following variations during a longer period of time after plant avoidance learning revealed that ND lip density transiently increased without calycal volumetric changes in the treated subcolony. **(E)** ND lip synapsin-IR boutons density and **(F)** lip volume at different times after incorporation of the untreated plant (*fir*_Ctrl,_ control). Incorporation of control leaves did not promote significant changes in the lip. Dots represent the mean value and solid lines the S.D. Horizontal dotted lines indicate mean bouton density quantified before treatment (day 0). Asterisks indicate significant differences between day 0 and different times after leaves incorporation into the fungus garden; *p < 0.05. Treatment: day 0, *N* = 5; day 2, *N* = 8; day 4, *N* = 8; day 15, *N* = 6. Control: day 0, *N* = 7; day 2, *N* = 7; day 4, *N* = 8; day 15, *N* = 8.

In accordance with the previous learning experiment, the foragers’ initial preferences for firethorn leaves (Figures 7A,B, day 0) was not modified in control subcolonies after incorporation of the untreated leaves (GH = 3.10, *p* = 0.54, *N* = 75, *df* = 4; G-test; Figure 7B). Ants from treated subcolonies showed a clear rejection behavior at the first day after incorporation, which was still observed 15 days thereafter (GH = 61.11, *p* < 0.0001, *N* = 106, *df* = 4; G-test; Figure 7A), denoting long-term avoidance memory beyond the time observed in the learning experiment 1.

Quantification of synapsin-IR boutons in the ND lip showed a transient variation in the bouton density after learning (*F*_3,23_ = 3.38, *p* = 0.036; ANOVA; Figure 7C). In agreement with the results found in learning experiment 1, synapsin-IR bouton density significantly increased 2 days after the incorporation of the treated leaves. Four days after treatment, density decreased showing intermediate values between days 0 and 2, and at day 15, densities had decreased to the initial state. These changes were modality-specific as no significant variations of synapsin-IR bouton densities were found in the visual collar (*F*_3,16_ = 1.72, *p* = 0.20; ANOVA). No significant volumetric changes were found in the lip (*F*_3,23_ = 1.68, *p* = 0.20; ANOVA; Figure 7D) and in the collar (*F*_3,23_ = 0.61, *p* = 0.61; ANOVA). This suggests that the changes in synapsin-IR bouton densities associated with long-term olfactory avoidance memory formation were due to an increase in MG densities and numbers in the ND lip followed by a decrease back to initial levels. In control ants, no variations in bouton densities (*F*_3,26_ = 0.38, *p* = 0.77; ANOVA; Figure 7E) or calyx volumes (lip: *F*_3,26_ = 1.93, *p* = 0.15; ANOVA; Figure 7F; collar: *F*_3,26_ = 2.87, *p* = 0.06; ANOVA) were found during all tested days after plant incorporation.

## Discussion

This study shows that long-term memory formation following avoidance learning is associated with a transient increase in the number of MG in olfactory regions of the MB calyx, while no changes were detected in visual subregions. This suggests that long-term memory formation leads to a modality-specific dynamic reorganization of synaptic complexes (MG). We hypothesize that new MG are formed and subsequently (most likely) others are eliminated. The results also show that these processes clearly differ from effects triggered by sensory exposure alone. Simultaneous collection of multiple non-harmful plant species with distinct chemical composition led to a marked decrease (pruning) in MG numbers in the olfactory lip.

### Changes After Sensory Exposure

Under natural conditions leaf-cutting ants typically harvest several plant species and incorporate all of them into the fungus garden simultaneously (Cherrett, 1989; Wirth et al., 2003). This means that they are exposed to a large variety of plant odors. Laboratory ants, in comparison, can be reared in a relatively deprived sensory environment and fed, for example, with only one or two plant species year round (e.g., blackberry and rose). A poor olfactory environment under laboratory conditions might also be a reason for the lack of brain microarchitectural differences between 1-month old individuals—still performing in-nest tasks or just becoming new foragers—and the outside worker group—composed by a high proportion of experienced ants, as we observed for the lip in the age-related plasticity control experiments. The fact that age-related volume changes in the controls were absent in the collar, however, may not rule out volume independent reorganization of visual MG associated with the transition to foraging.

When foragers of a laboratory subcolony of *A. ambiguus* were offered a high variability of suitable plant species over 3 days, the olfactory lip showed a marked decrease in bouton density. This suggests that enriched odor exposure alone leads to pruning of olfactory PN boutons. Synaptic pruning is a common refinement process in which axonal branches are eliminated in a competitive manner, leading to changes in synaptic structure and strength (Kantor and Kolodkin, 2003; Luo and O’Leary, 2005; Gogolla et al., 2007). A greater diversity of odorants from multiple plant species may result in higher neuronal activation in the olfactory pathway and, at the same time, more competition across a broader range of activated olfactory PNs. Although coding of complex mixtures and lateral interaction between PNs still is not well understood, it can be expected that enhanced competition among them may result in elimination of less used MGs compared to strongly used ones. Similarly to our results, distributed sensory pruning was also found in the visual collar of both ants and honeybees after sensory exposure by visual stimulation with broad light spectra (Stieb et al., 2010, 2012; Scholl et al., 2014). The decrease in MG numbers following sensory exposure was also shown to be associated with a massive outgrowth of Kenyon cell dendritic branches (Stieb et al., 2010). In leaf-cutting ants the marked MG reorganization after increased sensory exposure may be an important preparation of the olfactory MB microcircuits for subsequent associative learning and memory formation of the suitability of different plant species for the fungus garden.

### Changes After Avoidance Learning and Long-Term Memory Formation

Ants learned to avoid an initially accepted and preferred plant because of its harmful effects on the symbiotic fungus. Long-term memory formation was evidenced by a strong and specific rejection behavior to the plant as late as 15 days after the initial incorporation of fungicide-treated leaves, even though no further negative reinforcement had occurred. Avoidance learning has been shown to occur in several *Acromyrmex* and *Atta* leaf-cutting ant species in both field and laboratory. The effect is plant-species specific and is prompted by the presence of a harmful substance to the fungus in the leaves (which is not recognizable and harmless to the ants) or the presence of herbivore-induced plant compounds, and involves the formation of long-term olfactory memory (Ridley et al., 1996; North et al., 1999; Herz et al., 2008; Saverschek et al., 2010; Saverschek and Roces, 2011; Thiele et al., 2014). Delayed rejection behavior in treated subcolonies took place differentially 1 or 2 days after the initial incorporation of the CHX-infiltrated leaves into the fungus garden. The onset of this behavior might depend on different factors and it was reported to occur within 10 h or several days after the onset of foraging on treated substrates (Ridley et al., 1996; Herz et al., 2008).

Not all cues potentially involved in the learning process inside the nest are known. Even *naïve* workers with no previous experience with treated leaves in the foraging arena learn to avoid the plant associated with the harmful effects if they get in contact with the fungus within 1 or 2 days after the incorporation of leaves. Interestingly, *naïve* workers that interact with the fungus garden after 2 days do not reject the formerly-treated plant (Herz et al., 2008), indicating that either the fungicide-induced changes in the fungus are gone and/or no longer detectable, or the cues to identify the incorporated plant fragment cannot be recognized anymore. It is still an open question what kind of olfactory and/or gustatory cues from the fungus may trigger the avoidance response (North et al., 1999). Workers would be able to use multisensory stimuli to identify plant species, but plant odor is sufficient for *A. ambiguus* foragers to retrieve their avoidance memory (Saverschek and Roces, 2011). We therefore focused on the effects in the olfactory input region of the MB calyces. Indeed, neuronal changes were found exclusively in the olfactory lip, while no changes appeared in the visual collar after plant avoidance learning. Similarly, in honeybees it was shown that associative olfactory learning related plasticity affects MG organization in the olfactory lip, but not the visual collar (Hourcade et al., 2010). Light exposure, on the other hand, did only affect the MB collar, but not the MB lip synaptic organization (Stieb et al., 2010, 2012; Scholl et al., 2014).

In our experiments, leaf suitability was changed by infiltrating the leaf tissue with CHX, which acts as a fungicide by inhibiting protein synthesis. We therefore carefully considered whether it may also act as a potential inhibitor of mechanisms underlying long-term memory formation in the ants. In the case that ants would have ingested small amounts of CHX from the leaves during their harvesting or processing, it appears unlikely that the observed changes in the lip MG numbers were due to an effect of this substance on neuronal plasticity. Two lines of evidence support this. First, both the increase in MG numbers after 2 days and long-term memory formation were not impeded, indicating no inhibition effects during the first 2 days after harvesting of treated leaves. Second, MG numbers in the (visually innervated) MB collar remained stable over the observed period, even after 15 days. As modality specific effect of a protein synthesis inhibitor after ingestion is extremely unlikely, potential delayed effects on MG would be expected in both the lip and collar. For these reasons we rule out that the observed subsequent decrease in the lip MG numbers on days 4 and 15 after treatment was due to a potentially delayed effect of CHX.

### Long-Term Memory Associated Increase in MG Densities is Transient

A significant increase in MG boutons occurred 2 days after incorporation of harmful leaves, after the ants showed a clear avoidance response. In honeybees a similar increase in MG occurred after formation of a transcription-dependent olfactory long-term memory following appetitive associative learning (Hourcade et al., 2010). This suggests that both appetitive and aversive associative olfactory learning lead to an increase in MG densities in the olfactory region of the MB in social insects. Formation of new synapses also has been suggested to be a substrate for associative memory storage in mammals (Moser, 1999; Lamprecht and LeDoux, 2004).

If long-term memory formation would always be coupled to an increase in MG densities, this would likely be limited by physical constraints, in particular neuronal space. Our results show that the initial increase in MG density is followed by a decrease back to initial densities. The most likely mechanism underlying this process would be the formation of new MG (novel associative connections) followed by the elimination of (less used) others resulting in the reorganization of the associative network in the MB calyx. In other words, we propose that before learning occurs, MG densities in the lip region are pre-determined —at least in part— by the age and the previous sensory exposure of the individuals. Two days after olfactory learning new synaptic connections are formed increasing the number of MG. Then, several days after learning, other (less used) MG complexes are competitively eliminated leading again to a reduction in MG density to the initial densities (before learning), but a change in the functional connections of the associative neuronal network. The transient increase of MG in *A. ambiguus* may reflect this process of reorganization between novel boutons and subsequent pruning. The resulting change in the associative synaptic microcircuits may then preserve the memory trace until it is eliminated by strong competition. This mechanism would maintain MG densities (and energetic costs) at relatively constant levels and, at the same time, allow neuronal networks to preserve plasticity capacity for future memory formation. Whether this is likely to be a general mechanism for the storage of long-term memory in small insect brains needs to be further investigated in the future by looking more closely at odorant-specific circuits.

## Conflict of Interest Statement

The authors declare that the research was conducted in the absence of any commercial or financial relationships that could be construed as a potential conflict of interest.

## Abbreviations

CHX: cycloheximide
D lip: dense lip
HW: head width (mm)
M: ant mass (mg)
MB: mushroom body
MG: microglomeruli
ND lip: non-dense lip
PN: projection neuron.
